# Reducing Kidney Discard With Artificial Intelligence Decision Support: the Need for a Transdisciplinary Systems Approach

**DOI:** 10.1007/s40472-021-00351-0

**Published:** 2021-11-15

**Authors:** Richard Threlkeld, Lirim Ashiku, Casey Canfield, Daniel B. Shank, Mark A. Schnitzler, Krista L. Lentine, David A. Axelrod, Anil Choudary Reddy Battineni, Henry Randall, Cihan Dagli

**Affiliations:** 1 Engineering Management & Systems Engineering, Missouri University of Science & Technology, 223 Engineering Management 600 W 14th St, MO 65409 Rolla, USA; 2 Psychological Science, Missouri University of Science & Technology, Rolla, MO USA; 3 Saint Louis University Transplant Center, St. Louis, MO USA; 4 University of Iowa Transplant Center, Iowa City, IA USA

**Keywords:** Kidney discard, Artificial intelligence, Transdisciplinary, Systems science, Decision-making

## Abstract

**Purpose of Review:**

A transdisciplinary systems approach to the design of an artificial intelligence (AI) decision support system can more effectively address the limitations of AI systems. By incorporating stakeholder input early in the process, the final product is more likely to improve decision-making and effectively reduce kidney discard.

**Recent Findings:**

Kidney discard is a complex problem that will require increased coordination between transplant stakeholders. An AI decision support system has significant potential, but there are challenges associated with overfitting, poor explainability, and inadequate trust. A transdisciplinary approach provides a holistic perspective that incorporates expertise from engineering, social science, and transplant healthcare. A systems approach leverages techniques for visualizing the system architecture to support solution design from multiple perspectives.

**Summary:**

Developing a systems-based approach to AI decision support involves engaging in a cycle of documenting the system architecture, identifying pain points, developing prototypes, and validating the system. Early efforts have focused on describing process issues to prioritize tasks that would benefit from AI support.

## Introduction

**Fig. 1:**
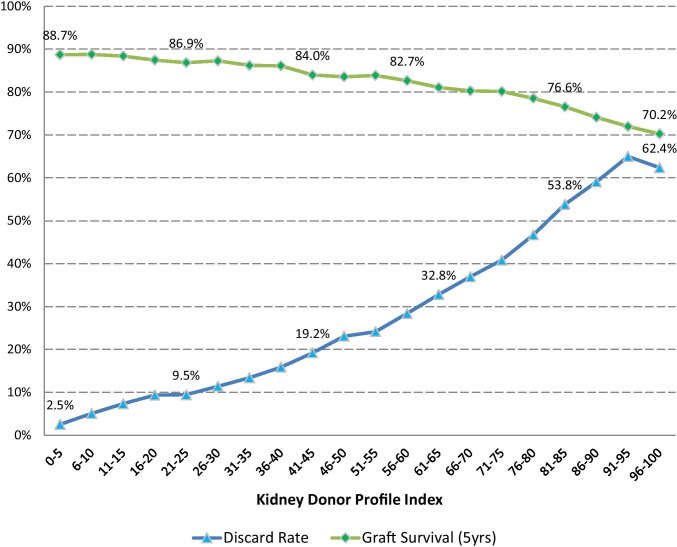
Kidney discard (dotted blue) increases for high Kidney Donor Profile Index (KDPI) organs, despite high rates of graft survival after 5 years (solid green) based on Scientific Registry Transplant Recipients (SRTR) data [1]

The demand for kidneys far outpaces supply. In the USA, nearly 150,000 people are on the waiting list for kidney transplants, but only 24,273 kidneys were transplanted in 2019 [1]. Notably, despite the large unmet need, 20% of procured deceased donor kidneys are discarded in current practice [2]. Even with lower quality organs, transplantation has been proven to be life-extending, cost-effective, and often cost-saving for appropriate candidates [3]. However, as shown in Fig. 1, the discard rate rises exponentially with measures of organ quality, such as higher Kidney Donor Profile Index (KDPI) scores [4]. The high discard rate for higher KDPI scores represents a substantial opportunity for increased kidney utilization, primarily from older donors with more comorbidity [5]. Artificial intelligence (AI) decision support may improve kidney utilization, if effectively designed to support clinician decision-making and provide better real-time access to data-driven predictions.

AI has been applied in many healthcare applications ranging from feature identification for radiology images [6] to prediction of clinical events from electronic health records [7] to classification for mental health diagnoses using social media data [8]. One popular approach to AI is deep learning, which is a subfield of machine learning that uses algorithms inspired by the structure of the brain called artificial neural networks. Essentially, the algorithms process data in layers to extract features. There can be hundreds of hidden layers or “neurons” between the inputs and outputs of the model. Advances in deep learning aim to increase accuracy while minimizing computations, maximizing speed, and reducing pre-processing requirements. For example, deep convolutional networks (also called DenseNet) increase the number of connections between layers in a feed-forward fashion to increase feature reuse [9]. A feature can be any measurable property within the data, similar to the explanatory variables used in linear regression. However, the features and number of layers are determined by the algorithm using the data, rather than by experts. Compared to statistical models, AI models can incorporate more data types (e.g., numerical, image, natural language) with fewer assumptions to improve prediction accuracy. In the context of transplant healthcare, United Network for Organ Sharing (UNOS) maintains extensive records associated with donor and recipient characteristics, donor-recipient matching, and patient outcomes [10]. AI models have the potential to speed up the discovery of insights from these types of large datasets to improve quality of care, reduce misdiagnosis, and optimize treatments.

However, AI systems can suffer from bias and make systematically poor judgments. For example, IBM’s Watson used natural language processing to analyze electronic health records and medical databases to make cancer treatment recommendations. MD Anderson agreed to test the prototype in their leukemia department and canceled the project after spending $62 million because it routinely gave clearly erroneous recommendations, reducing clinician trust [11]. In the context of criminal justice, AI models that predict the probability of recidivism for parole decisions tend to predict higher rates of reoffending for black people, contributing to systemic racism [12–14]. Even in cases where AI systems show improved outcomes, they can struggle with low adoption, a phenomenon known as algorithm aversion [15, 16••]. There is reason to believe that AI can improve healthcare, but it must be executed carefully to avoid negative outcomes.

To effectively integrate AI into transplant healthcare, a transdisciplinary systems approach is needed to design and evaluate the use of AI decision support systems in a participatory research framework. A transdisciplinary approach leverages knowledge and methods from engineering, social science, and the decision domain (transplant medicine) [17]. For complex systems, a systems approach provides tools and techniques for quantifying interactions between system elements to discover emergent properties [18]. Participatory research actively engages end-users, as well as decision-makers, early in the research process to build mutual trust within the community and identify potential barriers early [19]. This article highlights the promise and limitations of using AI decision support to motivate the use of a transdisciplinary systems approach. We frame this discussion in the context of our active application of this approach to the problem of kidney discard.

## Promise and Limitations of Artificial Intelligence Decision Support

An AI decision support system may be able to increase decision speed, reduce cognitive burden to improve decision outcomes, and facilitate communication between patients and clinicians. Although AI decision support systems have substantial potential, there is limited evidence of benefits. Most existing studies for AI systems are retrospective and have not been validated in a clinical setting [20•]. Integrating AI into healthcare may be particularly helpful for standardizing care when there is high heterogeneity and inconsistency between clinicians. The level of automation will vary depending on the decision context and operator expertise. Automation levels can vary from no assistance, to suggestions, to supervised operation, to unsupervised operation [21]. In many cases, the cost savings associated with AI systems are related to faster operations and reduced labor costs.

In the context of transplant care, researchers have developed models to predict graft outcomes and improve donor-recipient matching. In a 2020 review of 9 liver transplant models, the most common AI approach for predicting graft survival was artificial neural networks, and the number of input variables ranged from 10 to 276. When compared to more standard liver metrics [i.e., Model for End-Stage Liver Disease (MELD), balance of risk (BAR), and survival outcome following liver transplantation (SOFT)], multiple studies demonstrated that the AI predictions were more accurate [22••]. In a 2019 review of 14 kidney transplant models, decision trees and artificial neural networks were the most common and had the highest accuracy for predicting graft failure based on donor and recipient characteristics [23••]. However, another 2019 review of 7 kidney transplant models found mixed evidence that machine learning approaches exceeded the performance of traditional statistical models [24••].

Despite increasing evidence that AI models are more accurate, there has been little progress integrating these tools into transplant healthcare. We highlight three primary barriers, (1) overfitting and bias, (2) explainability, and (3) trust and ethical decisions, which limit the effectiveness of AI models and are being actively studied across engineering and social science disciplines to identify solutions.

### Overfitting and Bias

AI systems struggle with overfitting, particularly for unbalanced data. A model is overfitted when it closely matches training data and generates poor predictions for new scenarios. This is often attributed to the data insufficiency problem, where there is inadequate data to properly train a model or the data are highly unbalanced, which also reduces the number of training cases for an event of interest. In the context of kidney transplants, the decision to accept a kidney is a complex calculus and some key data are not available in the dataset (e.g., anticipated ischemic time, biopsy results, and recipient cardiac status). Similarly, a model can be biased if the data are biased (e.g., due to racial or gender discrimination) or the model systematically deviates from the data.

**Fig. 2:**
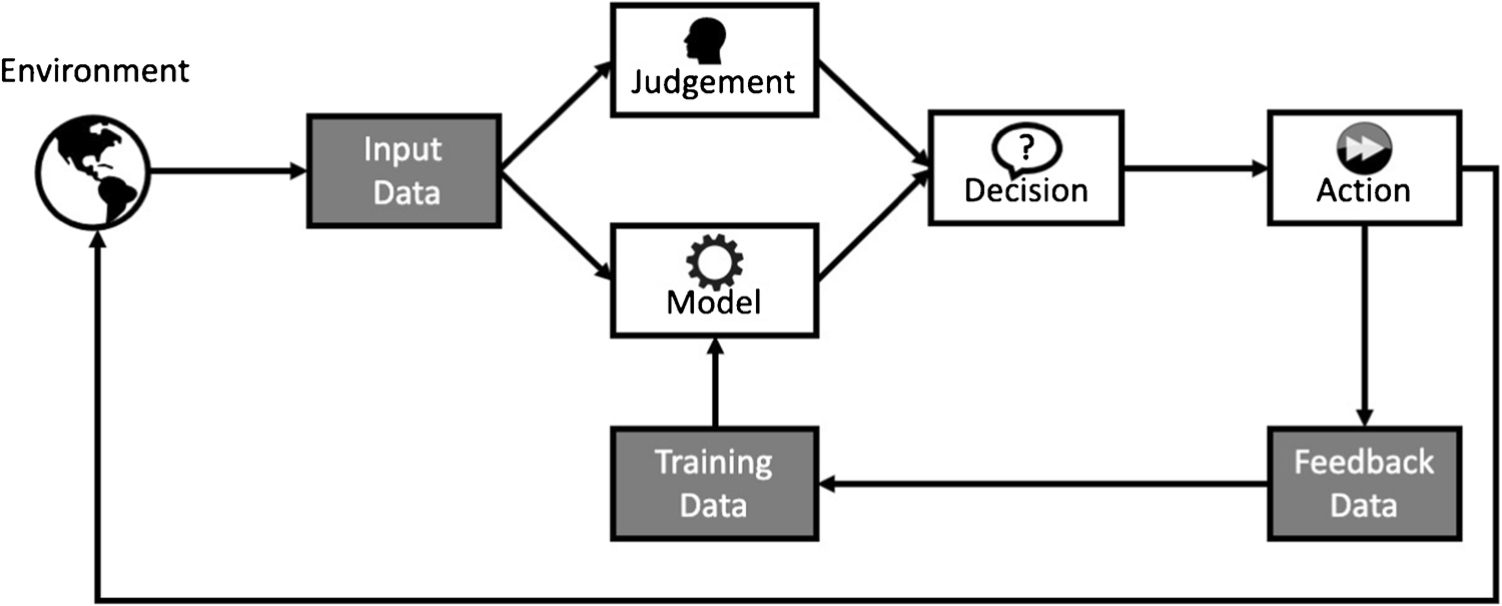
Summary of feedback loops from input and training data that allow a deep learning model to adapt over time

This limitation may be addressed via improved modeling techniques, training human operators to compensate, or some combination. Deep learning models are able to adapt to changing data over time, as shown in Fig. 2. The model output is influenced by both the input data and the training data. As decisions are made, those actions are used as feedback to train the model so it improves over time. For larger shifts, retraining models for new information is a time-consuming and labor-intensive process, but transfer learning and ensemble models can improve real-time predictions. Transfer learning, which only alters the final deep learning layers to accommodate new data and labels, is the most promising approach to rapidly accommodate incremental data changes [25]. Ensemble models are used to reduce the deep learning models’ variance and combine multiple predictions [26]. This is particularly valuable for fusing multiple stakeholder- or task-specific AI models into a higher-level model. However, regardless of the effectiveness of these techniques in theory, there will always be edge cases where the AI model makes poor predictions. In those cases, users need to be able to compensate for model limitations. This will likely require users to understand why a model might make mistakes and what type of information would be out of scope for the AI inputs. For example, when deciding whether to perform a transplant, transplant surgeons often consider information beyond what is available in the UNOS database, such as kidney imaging. When evaluating AI models, the historical record is assumed to be the ground truth, given the lack of available counterfactuals. However, researchers acknowledge that the historical record is likely biased and a research area focused on Fairness, Accountability, and Transparency (FAccT or FAT) is developing [27].

### Explainability

AI systems are unlikely to be adopted if they are perceived as black-boxes and difficult to use. The technology acceptance model suggests that adoption is based on the ease of use and usefulness [28], including for AI systems [29] in healthcare contexts [30]. Deep learning models are often perceived as a black-box, making it difficult for clinicians to understand how a model works and how trustworthy it is [31]. In addition, there will always be some measure of uncertainty inherent in the output of an AI model. That uncertainty may be aleatoric (e.g., due to chance, poor compatibility between organ and candidate), epistemic (e.g., measurement error, catch-all rejection reasons), and ontological (e.g., structural, model validation process does not align with model usage) [32].

Explainable models are able to communicate how and why a specific recommendation or outcome has been derived [33]. Deep learning models may include anywhere from several to hundreds of layers and are best known for extracting high-level abstract features. Developers can use feature relevance scores to quantify the contribution of a specific feature to the model’s prediction and communicate that information to the user via linguistic and numerical expressions [34]. However, in some cases, providing additional information can reduce accuracy and increase confidence [35]. Even experts are sensitive to cognitive biases and heuristics, such as confirmation bias, where users are more confident because the AI recommendation aligns with their initial perception, and anchoring effects, where users under (or over)-estimate the effect of choosing a different option because of anchoring on a provided number [36]. Experimental evidence is needed to ensure that the AI system’s communication has the desired effect [37]. For example, preliminary work on communicating uncertainty for deep learning models found that a confidence bar visualization had little effect on decision-making [38].

### Trust and Ethical Decisions

Even when there is a human-in-the-loop or in a supervisory role, people do not always trust or collaborate well with recommendations from AI systems. They may not want AI systems to make decisions dealing with ethics, including in medical contexts, even if the AI system is limited to an advisory role [39••]. Even if the AI system makes the same decision as a person, people tend to perceive the AI system as less trustworthy and more blameworthy [40]. In general, trust is reduced and the AI is perceived as morally wrong, if the AI system errs, is biased, or engages in unethical behavior [41].

The way an AI system is framed can enhance trust, even in the context of moral decisions. For example, anthropomorphism and perceptions of an AI having more mental capacity can increase trust [42] but can also enhance perceptions of it being morally wrong if it errs [43]. In addition, when users have the ability to make slight changes to the AI after it errs, those users continue to use it [44]. Trust, confidence, and ultimately use of a system can be highly eroded by the perception that an AI system has errors or is subject to potential errors or bias.

## Value of a Transdisciplinary Systems Approach

A transdisciplinary systems approach solves complex problems that occur at the interfaces between disciplines and limit the implementation of new technologies across domains. These domains can be decomposed as a system of systems, with multiple parts that work together and are independent systems in their own right, such as aerospace manufacturing [45]. This is particularly important for complex adaptive systems, which evolve and adapt over time, such as disease dynamics [46]. By conceptualizing transplant healthcare using these systems theories, we can leverage techniques for documenting existing and future states to support the introduction of new technologies, like AI.

Transdisciplinary science is shifting how we scope problems, create models, and evaluate interventions. For example, adequately modeling food and nutrition security requires integrated models of the environment (e.g., to determine impacts of pollution on soil quality), agricultural systems (e.g., to estimate food availability), public health (e.g., to predict malnutrition), and individual behavior (e.g., to anticipate demand for certain types of food). Modeling these aspects separately fails to address the challenges that occur at the interfaces (e.g., increased demand for fresh food may lead to unsustainable farming practices, which further reduces cultivatable land) [47]. Systems dynamics and agent-based modeling techniques are particularly effective for supporting these types of integrated simulations [48]. However, transdisciplinary research is only possible with interdisciplinary teams that bring together their siloed theories, data collection methods, and analytical techniques from engineering, social science, and the humanities into a holistic conceptual framework. In addition, by including domain experts and users in the team, both academics and practitioners benefit from more relevant research, capacity building, and increased potential for implementation [49].

To this end, model-based systems engineering (MBSE), first introduced in 1993, has developed techniques to document and visualize complex systems to identify requirements and anticipate emergent properties of complex systems. MBSE uses formal models to document a system and replaces the traditional document-based approach to product development. This approach increases consistency and scales with complexity for system specification, design, and validation. These methods rely on a system architecture, which centralizes system documentation by articulating various views to represent the “sole source of truth” [50]. The most popular modeling language is Systems Modeling Language (SysML) [51], which can be visualized by Cameo Systems Modeler software [52]. First developed in 2003, SysML is used to describe systems (i.e., making pictures) as well as support an executable system architecture (i.e., running simulations). It is primarily used for design and validation of systems, rather than implementation. In the design phase, SysML models can improve interoperability and support complex concurrent design processes. In the validation phase, SysML models can support evaluation of the effect of a new technology after implementation [53••]. These tools have been extensively adopted in the defense and aerospace industries to guide complex engineering design projects [54]. However, systems architecting practices have historically approached human behavior as an afterthought. MBSE tools can facilitate communication between disciplines by documenting and translating between the different perspectives (e.g., engineers and psychologists) [55]. In the context of designing for human–machine teams, a recent SysML extension articulates the roles and responsibilities of human versus machine team members and supports interdependence analysis to evaluate teamwork outcomes [56•]. There is increasing interest in applying these techniques to other complex problems, especially in the context of healthcare [57, 58].

## Applying a Transdisciplinary Systems Approach to Reduce Kidney Discard

**Fig. 3:**
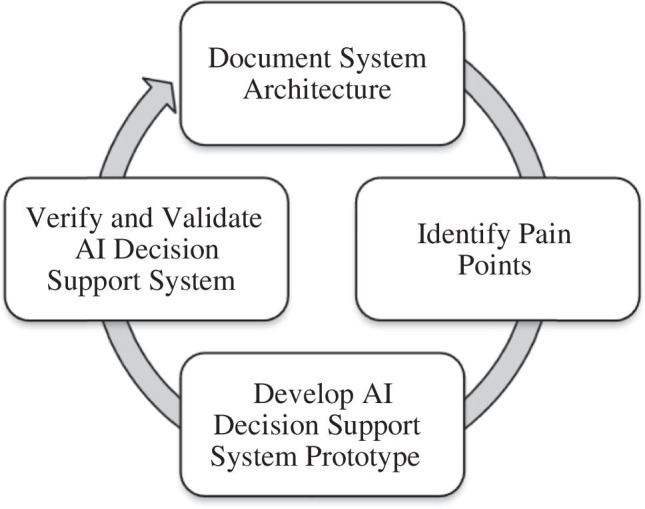
A transdisciplinary systems approach is supported by a cyclical development process for an AI decision support system

We are currently engaged in ongoing work to apply a transdisciplinary systems approach to the design and implementation of an AI decision support system to reduce kidney discard. As summarized in Fig. 3, this approach is developed through a cyclical process wherein we (1) document the current system architecture, (2) identify pain points that would benefit from AI support, (3) develop prototypes of an AI decision support system, and (4) verify and validate the AI system to ensure we achieve desirable outcomes. An iterative cyclical process incorporates multiple opportunities for stakeholder engagement that benefit from a “fail fast” philosophy.

**Fig. 4:**
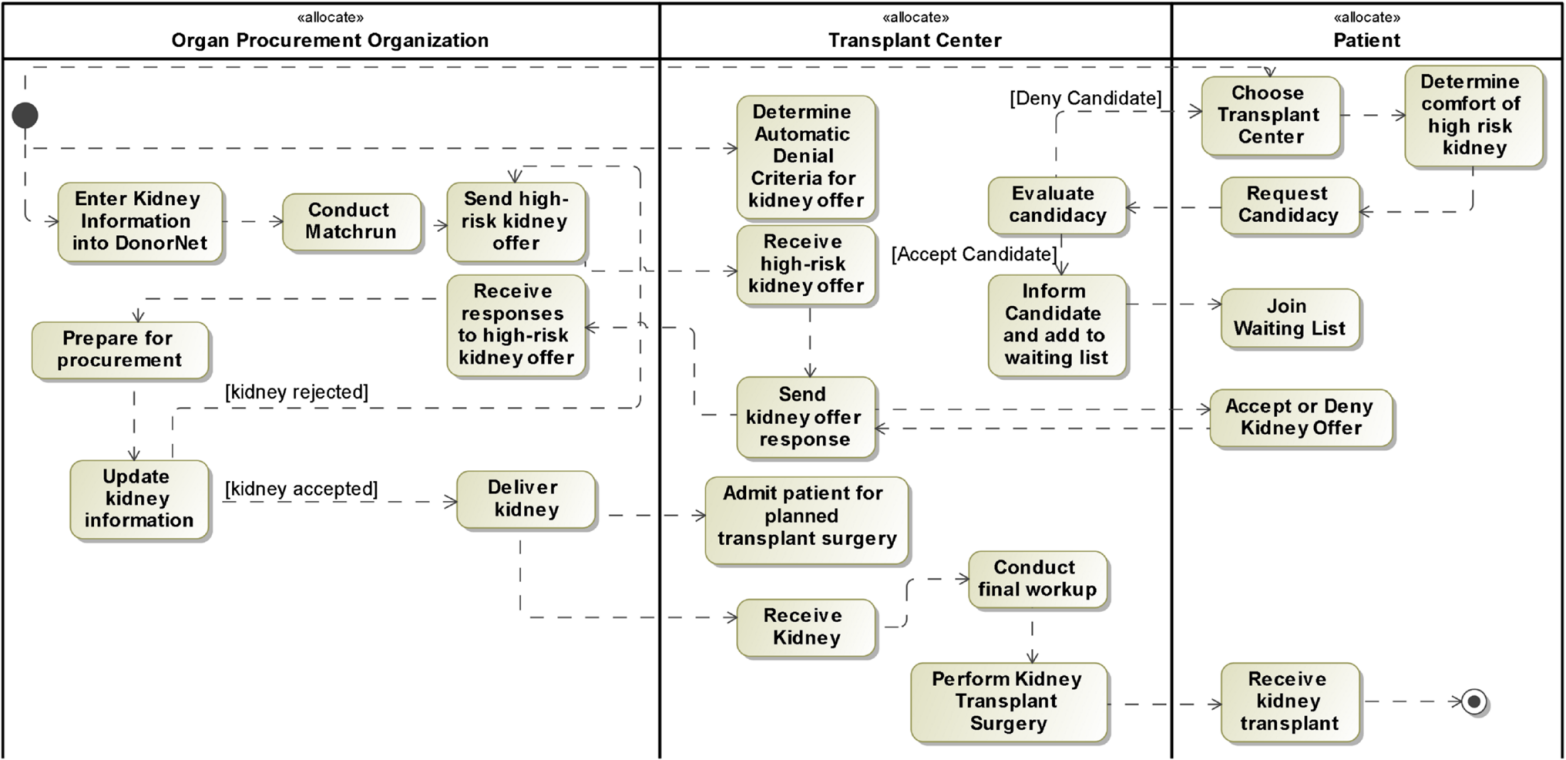
SysML activity diagram or workflow for kidney allocation based on (a) organ procurement organization, (b) transplant center, and (c) transplant patient views

Based on interview data, we have developed a SysML activity diagram to represent the workflow, which is one aspect of the organ allocation system architecture, across and between these stakeholder groups (see Fig. 4). In the transplant context, key stakeholders include organ procurement organizations (OPO), transplant centers, and patients. In addition, we have identified six major process issues in the kidney transplant workflow that could be alleviated via support from an AI decision support system. Much of the work to place less desirable (“lower quality”) kidneys occurs after midnight (process issue 1: environmental stress). Transplant centers receive organ offers via DonorNet and have 1 h to evaluate the offer and enter a decline or provisional acceptance of the offer (2: time pressure). Often, transplant centers will provisionally accept an offer to keep their options open, even if there is a low likelihood that they will ultimately accept the offer (3: local optimization). The transplant team receives access to extensive information including the donor’s medical history, known risk factors for organ function (e.g., age, cause of death, diabetes, hypertension, hepatitis C infection status, KDPI). After the kidney is procured, transplant center staff can adjust their decision as more information becomes available or based on patient input and compatibility (4: evolving information). Ultimately, the surgeon has until the moment of transplantation to decide to decline a kidney offer. Offers that are rejected at this stage are at the highest risk of discard and very difficult to re-allocate.

When evaluating kidney offers, transplant teams must assess the benefits and costs of waiting for a higher quality kidney versus moving forward with a lower quality kidney. In addition, when deciding whether to perform a transplant with a given donor, surgeons consider many factors, ranging from medical compatibility to assessment of complex donor clinical information including imaging and other diagnostics, which can all affect the success of the transplant (5: complex decision space). Further, transplant centers, and individual surgeons, vary in their ability to care for patients with complications (e.g., patients who get hepatitis C infections from donors) and risk posture based on recent failed transplants (6: heterogeneous risk posture). All of these process issues interact with each other to increase the complexity of addressing kidney discard via an AI decision support tool.

In addition to articulating the current system architecture, we are relying on stakeholder input to prioritize which tasks to focus on for an AI decision support system. In the context of online workshops, we engaged small groups of transplant stakeholders to organize tasks in an effort vs. impact prioritization matrix. Focusing on the steps that are high effort to execute and high impact in affecting kidney discard, the top candidates for an AI decision support system include (1) OPO efforts to reallocate denied kidneys and (2) transplant center/patient decision-making to accept/deny high-risk kidney offers.

## Conclusions

AI systems can improve healthcare delivery, but it is challenging to design an effective decision support system. In the context of transplant healthcare, kidney discard is a complex problem that will require coordination across stakeholders and shifting clinician behavior. An AI decision support system could support efforts to better leverage real-time data-driven decision-making during this transition toward increased kidney utilization. However, AI systems frequently suffer from overfitting, poor explainability, and low user trust, reducing the likelihood of widespread adoption. Researchers in engineering and social science disciplines are actively identifying strategies to improve human–machine interaction for these types of systems. For overfitting, promising strategies include the use of transfer learning and ensemble models as well as improved training to allow human users to compensate for model limitations. Deep learning models can generate feature relevance scores to communicate how specific features contribute to a prediction, but experimental evidence is needed to determine if these communications have the desired outcomes. Trustworthy models tend to use anthropomorphism to increase perceptions of the AI having mental capacity or allow users to make small changes to the model after it errs. However, there have been few opportunities to examine the effect of combining these approaches in a real-world clinical environment.

Using a transdisciplinary systems approach in a participatory research framework, we can iteratively refine an AI decision support system design to maximize potential effectiveness. Transdisciplinary collaborations support the development of both the operation and interface of an AI decision support system in tandem. Rather than solving a challenge like overfitting with technology alone, there are opportunities to leverage human expertise to increase the system-level effectiveness. Similarly, early engagement of domain experts (e.g., transplant stakeholders) can increase trust and the likelihood of developing an implementable system. A system architecture is useful for visualizing the system for cross-discipline conversations and solution development.

Based on ongoing work to develop an AI decision support system to reduce kidney discard, we outline a cyclical approach for designing and testing the system design that involves (1) documenting the system architecture, (2) identifying pain points, (3) developing prototypes, and (4) validating the system. Based on stakeholder input, we have identified six system characteristics that will inform the design, including environmental stress, time pressure, local optimization, evolving information, complex decision space, and heterogeneous risk posture. We are developing prototype AI systems for (1) OPO efforts to reallocate denied kidneys and (2) transplant center decisions to accept/deny high-risk kidney offers [59]. This approach is time consuming and requires extensive stakeholder engagement. But ultimately, this will lead to a better product and better outcomes for patients.
